# Chemical and genetic characterization of lipopeptides from *Bacillus velezensis* and *Paenibacillus ottowii* with activity against *Fusarium verticillioides*

**DOI:** 10.3389/fmicb.2024.1443327

**Published:** 2024-08-26

**Authors:** Gisele de Fátima Dias Diniz, José Edson Fontes Figueiredo, Kirley Marques Canuto, Luciano Viana Cota, Ana Sheila de Queiroz Souza, Maria Lúcia Ferreira Simeone, Sylvia Morais de Sousa Tinoco, Paulo Riceli Vasconcelos Ribeiro, Lourenço Vitor Silva Ferreira, Mikaely Sousa Marins, Christiane Abreu de Oliveira-Paiva, Vera Lúcia dos Santos

**Affiliations:** ^1^Department of Microbiology, Federal University of Minas Gerais, Belo Horizonte, MG, Brazil; ^2^Molecular Biochemistry Laboratory, Embrapa Maize and Sorghum, Sete Lagoas, MG, Brazil; ^3^Multiuser Laboratory of Chemistry of Natural Products (LMQPN), Embrapa Tropical Agroindustry, Fortaleza, CE, Brazil; ^4^Phytopathology Laboratory, Embrapa Maize and Sorghum, Sete Lagoas, MG, Brazil; ^5^Agrochemistry Laboratory, Embrapa Maize and Sorghum, Sete Lagoas, MG, Brazil; ^6^Molecular Biology Laboratory, Embrapa Maize and Sorghum, Sete Lagoas, MG, Brazil; ^7^Federal University of São João del-Rei, São João del Rei, MG, Brazil; ^8^Department of Agricultural Microbiology, Federal University of Lavras, Lavras, MG, Brazil; ^9^Soil Microbiology and Biochemistry Laboratory, Embrapa Maize and Sorghum, Sete Lagoas, MG, Brazil

**Keywords:** biocontrol, maize (*Zea mays* L.), lipopeptides, *Paenibacillus ottowii*, *Bacillus velezensis*, *Fusarium verticillioides*

## Abstract

**Introduction:**

The fungus *Fusarium verticillioides* significantly threatens maize crops in tropical soils. In light of this, biological control has emerged as a promising strategy to reduce fungicide costs and environmental risks. In this study, we aimed to test the antifungal activity of cell-free supernatant (CFS) from three *Bacillus velezensis* (CT02, IM14, and LIS05) and one *Paenibacillus ottowii* (LIS04) against *F. verticillioides*, thereby contributing to the development of effective biocontrol measures.

**Methods:**

The research employed a comprehensive approach. The antifungal activity of the bacterial strains was tested using cell-free supernatant (CFS) from three *Bacillus velezensis* (CT02, IM14, and LIS05) and one *Paenibacillus ottowii* (LIS04). The UPLC-MS evaluated the CFS to identify the main bioactive molecules involved in the inhibitory effect on *F*. *verticillioides*. Scanning electron microscopy (SEM) was used to assess the impact of CFS on spores and hyphae, and genome sequencing was conducted to identify the genes involved in biological control. These robust methodologies ensure the reliability and validate our findings.

**Results:**

The CFS of the four strains demonstrated significant inhibition of fungal growth. The UPLC-MS analysis revealed the presence of lipopeptides with antifungal activity, including surfactin and fengycins A and B expressed by the three strains of *Bacillus velezensis* and iturin A expressed by strains LIS05 and IM14. For *Paenibacillus ottowii*, fusaricidins, ABCDE, and five previously unreported lipopeptides were detected. Scanning electron microscopy (SEM) showed that treatments with CFS led to significant distortion and breakage of the *F*. *verticillioides* hyphae, in addition to the formation of cavities in the membrane. Genome mining confirmed the presence of genes coding for the lipopeptides identified by UPLC-MS, including the gene for iturin in CTO2. Genomic sequencing revealed that CT02, IM14, and LIS05 belong to different strains of *Bacillus velezensis*, and LIS04 belongs to *Paenibacillus ottowii*, a species recently described.

**Discussion:**

The four bacterial strains, including three novel strains identified as *Bacillus velezensis* and one as the recently described species *Paenibacillus ottowii*, demonstrate significant potential as biocontrol agents for managing fungal disease. This finding underscores the novelty and potential impact of our research.

## 1 Introduction

Maize is one of the most versatile crops, with various uses ranging from human consumption in natural or processed forms to animal feed and high-technology industries, such as pharmaceuticals, bioplastics, biopolymers, and ethanol production (Erenstein et al., 2022). Pathogenic fungi can cause plant disease and produce toxins that accumulate in grains, making the crop product unsuitable for human and animal consumption (Borràs-Vallverdú et al., 2022).

Fungal diseases constitute one of the main challenges for corn productivity in tropical climates. The fungus *Fusarium verticillioides* (Saccardo) Nirenberg is one of the most common pathogens associated with the maize crop in Brazil (Gomes et al., 2020; Sousa et al., 2022). This fungus infects the maize plants at all stages of development, causing seedling decay, stalk rot, ear rot, and grain contamination by mycotoxins (Murillo-Williams and Munkvold, 2008). Yield loss caused by *Fusarium* stalk rot of maize ranges from 10% in areas with low incidence to 30%−50% in severely affected areas. In contrast, *Fusarium* ear rot, characterized by discolored and a reduced number of grains, not only reduces yield but also influences the quality of the seeds (Li et al., 2010; Gai et al., 2018; Xu et al., 2023).

The intensive use of chemical fungicides for disease control has generated significant concern due to environmental damage and human and animal health (Fisher et al., 2018). Recently, using bacteria to control fungal pathogens has been recognized as a feasible, environmentally friendly, and low-cost approach for plant disease management (Tariq et al., 2020; Diniz et al., 2022). Bacteria possess multiple mechanisms for controlling fungal diseases, and several bacterial-based products have been registered and marketed as biopesticides (Bonaterra et al., 2022).

Among the bacterial groups used as biological control, *Bacillus* sp. and *Paenibacillus* sp. produce secondary metabolites cyclic lipopeptides with antifungal activity, such as iturin, fengycin, surfactin (*Bacillus* sp.), and fusaricidin (*Paenibacillus* sp.) (Shu et al., 2002; Sarangi and Ramakrishnan, 2016). Lipopeptides are secondary metabolites produced by bacteria and fungi (Ongena and Jacques, 2008). Their amphiphilic structure (lipid connected to peptide) interacts with cellular membranes, modifying their configuration and leading to cell lysis and death (Munusamy et al., 2020). Lipopeptides often exhibit potent antimicrobial activity and can inhibit fungal growth by disrupting cell membrane integrity (Sarwar et al., 2018; Tucuch-Pérez et al., 2018).

The identification of antifungal molecules is crucial for designing suitable strategies aiming at the development of bioproducts in the context of sustainable agriculture (Santos et al., 2023). Different approaches can be implemented to achieve the biochemical profile of a desired microorganism, including gas chromatography or liquid chromatography hyphenated with mass spectrometry techniques, metagenome, and genome-mining (Fu et al., 2019). These approaches also allow the finding of new bioactive molecules.

In this work, the cell-free supernatant (CFS) of three strains of *Bacillus velezensis* and one *Paenibacillus ottowii* were tested for antagonistic activity against *F*. *verticillioides*; UPLC-ESI-QTOF-MS identified the antifungal lipopeptides, and the complete genome sequencing showed that the three *Bacillus* (CT02, IM14, and LIS05) represent different strains of *Bacillus velezensis* and LIS04 belongs to *Paenibacillus ottowii*.

## 2 Material and methods

### 2.1 Microbial strains

In this study, the antifungal activity of cell-free supernatant (CFS) of two epiphytic bacterial strains previously isolated from maize (*Bacillus velezensis*, IM14, and CT02) and two from sorghum (*Paenibacillus ottowii* LIS04, and *Bacillus velezensis* LIS05) was tested against the phytopathogenic strain (CML 2778) of *Fusarium verticilioides* (Diniz et al., 2022, 2023). Chromatographic information on the antifungal lipopeptides and identifying the genes coding for lipopeptides by complete genome sequencing are provided.

### 2.2 Selection of medium for production and detection of secondary metabolites with antifungal activity

The four bacterial strains were grown in Trypticase Soy Broth (TSB), Potato Dextrose Broth (PDB), Luria-Bertani (LB) with glucose (20 g.L^−1^), and Landy medium (LM) to determine the best medium for the production of secondary metabolites with antifungal activity. After incubation at 28°C at 160 rpm for 72 h, each supernatant was collected by centrifugation at 9,000 rpm for 10 min (Hettich Universal 320 R Centrifuge, Tuttlingen, Germany), filtered through 0.22 μm membranes, and used in *in vitro* biological tests.

### 2.3 Broth microdilution susceptibility test

The cell-free supernatant's (CFS) antifungal activity was evaluated using the method described in the NCCLS document M38-A (CLSI - Clinical Laboratory Standards Institute, 2002). The plates were a 96-well U-bottom microdilution plate (Costar, Corning, USA) containing CFS at concentrations ranging from 0.3 to 100% (w.v^−1^) and negative (without fungus) and positive (with fungus) controls.

The spores of *F. verticillioides* were obtained from a fungal culture grown for seven days at 25°C, photoperiod of 12 h light in PDA. A spore suspension was filtered with cheesecloth to remove any mycelial fragments. The final spore concentration was adjusted to 1 × 10^4^ spores/ml by counting in a Neubauer chamber. A solution of *F. verticillioides* spores was added to each test well containing CFS, and the microplates were incubated at 25°C without shaking for 96 h. After this period, the absorbance reading was performed at 490 nm using a digital spectrophotometer (Biospectro SP22, São Paulo, Brazil). Each microplate was considered a replica, and the test was performed in triplicate. The Tucuch-Pérez et al. (2018) formula calculated the inhibition percentage:


$$\begin{array}{l} \%\;growth=\frac{A-B}{C}\;\left(100\right) \end{array}$$

Being: A = absorbance of treatment; B = absorbance of negative control; C = absorbance of positive control; and the formula (100 – % growth) determined the percentage of inhibition.

### 2.4 Effect of cell-free supernatant on *F*. *verticillioides* by scanning electron microscopy

The effect of CFS of bacterial cultures in TSB medium on the structural morphology of spores and hyphae of *F. verticillioides* was visually evaluated by ultra-high resolution scanning electron microscopy images. A spore suspension of *F. verticillioides* and the CFS, obtained as described above, were mixed in equal parts (1:1), incubated for 24 h at 25°C, and photoperiod of 12 h light. The control consisted of a TSB medium mixed with a spore suspension of *F. verticillioides*. After this time, the samples were fixed in Karnovsky solution (Karnovsky, 1965) by mixing in a 1:1 ratio, and the samples were stored at 4°C for 24 h. The coverslips were prepared by applying 5 μl of poly-L-lysine (0.1%) and adding 20 μl of each sample once the poly-L-lysine had dried. The samples were then washed three times in cacodilate buffer for 10 min each wash, followed by dehydration in acetone 25%, 50%, 75%, 90%, and 100%, with one wash for 10 min for each concentration up to 90 and three washes in 100% acetone. Next, the samples were dried at a critical point [CPD 030 - (Bal - Tec)] and metalized in a gold evaporator [SCD 050 - (Bal - Tec)]. The observations were done in a Scanning Electron Microscope – SEM - FEG ultra-high resolution, field-free, TESCAN Inc. CLARA model (Brno, Kohoutovice, Czechia).

### 2.5 Identification of secondary metabolites by UPLC-MS

For the separation of metabolites, each bacterium was grown in the culture medium with the highest antifungal activity, as determined by the microdilution test. After incubating pure colonies at 28°C and 160 rpm for 72 h, the supernatant was collected by centrifugation at 9,000 rpm for 10 min (Hettich Universal 320 R Centrifuge, Tuttlingen, Germany). The compounds were extracted by acid precipitation, adding 6M HCl until the pH was reduced to 2.0. Then, the acidic supernatants were stored in the refrigerator for 24 h and then centrifuged at 9,000 rpm for 10 min, and the pellets were dried in a cold freeze-dryer (Heto Lab Equipment, Allerød, Denmark). Chromatographic analysis was performed on an Acquity UPLC system coupled to a Quadrupole/Time of Flight (UPLC-ESI-QTOF-MS) Xevo System (Waters Corp, Milford, MA, USA). High-resolution mass conditions were processed according to the methodology described earlier by Souza et al. (2018), which was validated following the guidelines established by the International Conference Harmonization of Technical Requirements for Registration of Pharmaceuticals for Human Use (Singh, 2015) and the Brazilian Ministry of Agriculture Livestock and Food Supply Brazil (Souza et al., 2018). Chromatographic runs were performed on a Waters Acquity UPLC BEH column (150 x 2.1 mm, 1.7 μm). The fixed temperature was 40°C, mobile phases water with 0.1% formic acid (A), and acetonitrile with 0.1% formic acid (v.v^−1^) (B). The gradient ranged from 2 to 95% B (15 min), the flow rate was 0.4 mL/min, and the injection volume of 5 μl.

The ESI+ mode was acquired in the range of 110–1180 Da, source temperature of 120°C, desolvation temperature of 350°C, desolvation gas flow of 500 L.h^−1^, and capillary voltage of 3.2 kV. Leucine enkephalin was used as a lock mass, and the acquisition mode was MS. The instrument was controlled by Masslynx 4.1 software (Waters Corporation, Milford, MA, USA).

### 2.6 Data processing and multivariate analysis

Multivariate statistical analyses were used to reduce the complexity of the chemical data. Initially, the data acquired by the UPLC-QTOF-MS (raw format) were converted to the Analysis Base File (.abf) format using the Abf Converter software (http://www.reifycs.com/AbfConverter) and, later, were processed using the MS-DIAL v. 4.70 (Tsugawa et al., 2015). The processing parameters were: mass range *m/z* 50–1,180; MS1 tolerance of 0.01 and MS2 tolerance of 0.05; minimum peak height of 50; mass slice width of 0.1 Da; linear-weighted moving average as a smoothing method, using level 3 and minimum peak width of 5; sigma window value of 0.5 for deconvolution, and peak alignment, tolerance of 0.05 min and MS1 of 0.01 Da. The aligned data matrix for the 30 characterized compounds was exported from MS-DIAL as a .txt file and loaded into the Metaboanalyst 5.0 platform, following the standard protocol described by Chong et al. (2018). Initially, samples were normalized by sum and data scaled by Pareto scaling. Supervised analysis of partial least squares-discriminant analysis (PLS-DA) was performed to obtain the maximum separation between groups and predict which variables were discriminating for antifungal activity through the analysis of the Variable of Importance in Projection (VIP) > 1.0 (Selegato et al., 2022).

### 2.7 Sequencing and analysis of the complete genome of antagonistic bacteria

Genomic DNA extraction of *Bacillus velezensis* (IM14, LIS05, and CT02) and *Paenibacillus ottowii* (LIS04) was performed using the Wizard Genomic DNA Purification Kit (Promega, USA) and quantified on the Qubit ^®^ 2.0 fluorometer (Life Technologies). The genomes were sequenced on the Illumina HiSeq 4000 platform (Illumina, San Diego, CA, USA) at Beijing Genomics Institute—BGI (Shenzhen, China), using the 150-paired end strategy.

The sequenced reads were prepared for assembly using the Illumina NGS data program Trimmomatic (Galaxy Version 0.38.0). After trimming, the quality of the reads was verified using the FastQC software (Galaxy Version 0.73+galaxy0), eliminating reads with a quality lower than 20 in the Phred quality index. Next, the reads were assembled using the SPAdes software, and the assembly quality and genome completeness analysis was performed using the QUAST (https://quast.sourceforge.net/quast) and BUSCO (Benchmarking Universal Single-Copy Orthologs—https://gitlab.com/ezlab/busco), respectively (Noori et al., 2021).

The pre-assembled genomic sequences were annotated using PROKKA version 1.8 (Seemann, 2014) and RAST version 2.0 (Rapid Annotation using Subsystem Technology) software (Aziz et al., 2008). The assembled genomes were deposited in the GenBank database with the following accession numbers: JAUUUW000000000 (CT02), JAUUUY000000000 (IM14), JAUUUZ000000000 (LIS05), and JAUUUV000000000 (LIS04).

## 3 Results

### 3.1 Antifungal activity of secondary metabolites produced by bacteria

The four bacterial strains (LIS04, LIS05, CT02, and IM14) showed antifungal activity against *F*. *verticillioides* in the four culture media tested (Table 1).

**Table 1:** Inhibition percentage of *Fusarium verticillioides* by different concentrations of cell-free supernatant of bacterial strains grown in TSB, PDB, LB+G, and Landy media.

**Medium**	**Concentration of cell-free supernatant (%)**										**MIC**
	**Bacteria**	**0.3**	**0.7**	**1.5**	**3.1**	**6.2**	**12.5**	**25**	**50**	**100**	
TSB	LIS04	4.1	4.1	4.3	4.3	4.3	5.1	6.4	15.0	53.9	100
	LIS05	21.2	35.1	39.5	42.0	43.6	46.6	47.0	50.6	76.8	50
	CT02	45.3	53.1	53.3	55.4	58.9	60.5	61.4	61.8	84.6	0.7
	IM14	27.8	41.4	49.8	51.2	54.3	56.9	57.0	61.0	85.5	3.1
PDB	LIS04	2.3	2.6	2.6	5.0	9.2	17.6	28.7	48.9	60.0	100
	LIS05	13.9	18.6	30.6	38.0	41.9	47.4	62.2	64.5	75.7	25
	CT02	1.0	5.3	10.0	11.7	22.2	33.5	49.0	55.6	81.0	50
	IM14	0.0	4.6	14.1	21.5	30.7	40.1	43.8	81.7	97.4	50
LB + G	LIS04	6.2	6.9	8.1	9.4	9.7	11.1	11.3	26.9	62.5	100
	LIS05	26.6	27.8	36.8	37.3	43.0	43.4	48.4	57.7	63.4	50
	CT02	14.5	24.0	24.2	25.5	26.3	27.9	39.9	58.6	65.3	50
	IM14	15.4	17.9	19.4	23.0	23.3	23.3	45.6	63.1	71.5	50
Landy	LIS04	8.4	9.8	11.2	11.6	11.6	19.9	33.6	44.9	76.1	100
	LIS05	38.2	44.7	49.9	52.7	59.1	60.4	66.4	98.7	100	3.1
	CT02	35.5	44.8	50.9	55.7	61.3	67.7	70.2	80.5	96.7	1.5
	IM14	36.3	43.5	51.1	57.8	60.6	64.5	76.5	100	100	1.5

MIC, Minimum inhibitory concentration; TSB, Trypticase Soy Broth; PDB, Potato Dextrose Broth; LB+G, Luria-Bertani with glucose.

The antifungal activity of the four CFS evaluated by the microdilution plate method revealed the minimum inhibitory concentration (MIC), which inhibits the growth of *F*. *verticillioides* by at least 50% (Table 1). Each isolate's lowest inhibitory concentration of CFS showed significant variation among the strains and the culture media. The lowest inhibitory values were those produced by the CT02 strain grown in the TSB medium (0.7%) and the strains IM14 (1.5%) and LIS05 (3.1%) grown in the Landy medium. The CFS of the medium where each bacterium showed high antifungal activity was chosen for the UPLC-QTOF-MS analysis. For the isolate LIS04, regardless of the four culture media, a CFS concentration of 100% was necessary to inhibit at least 50% of the *F*. *verticillioides* growth. Thus, for the UPLC-QTOF-MS analysis, LIS04 was grown in the PDB medium, which was the medium where strain LIS04 showed 48.9 inhibition activity.

### 3.2 Scanning electron microscopic observations

**Figure 1:**
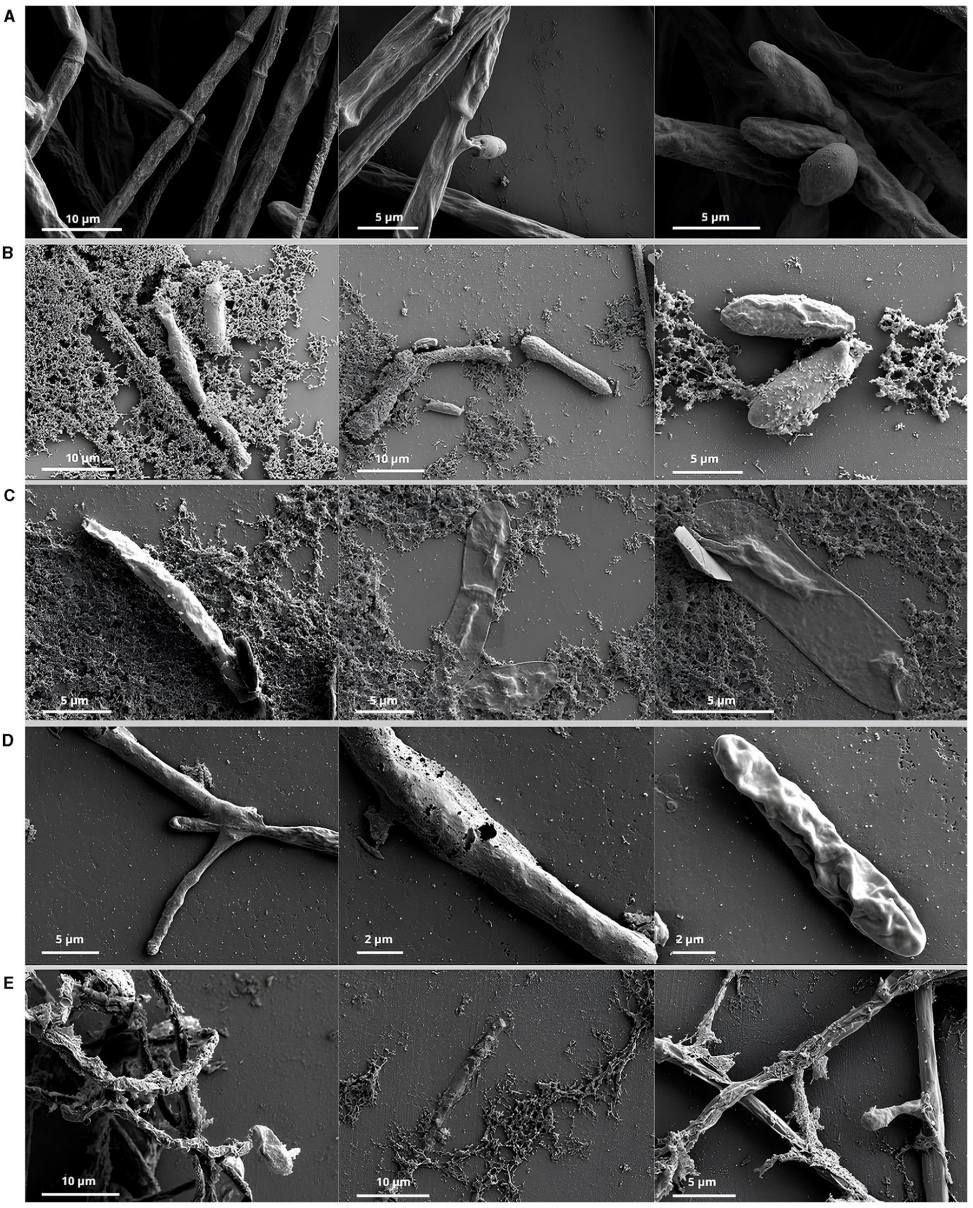
Scanning electron microscope of hyphae and spores of *Fusarium verticillioides*. Control: hyphae of *Fusarium verticillioides* developed from the germination of spores that had no contact with the CFS **(A)**. Hyphae originated from spore germination after treatments with the *Bacillus* sp. supernatant of CT02 **(B)**, IM14 **(C)**, *Paenibacillus* sp. LIS04 **(D)**, and *Bacillus* sp. LIS05 **(E)**.

Scanning electron microscopy observations showed healthy *Fusarium verticillioides* hyphae compared to hyphae germinated from spores treated with CFS (Figure 1). The hyphae were entire, smooth, and uniformly thick in all control samples (Figure 1A), compared to those in contact with the CFS, which were dehydrated, distorted, broken, and forming cavities (Figures 1B–E).

### 3.3 Identification of secondary metabolites by UPLC-MS

**Figure 2:**
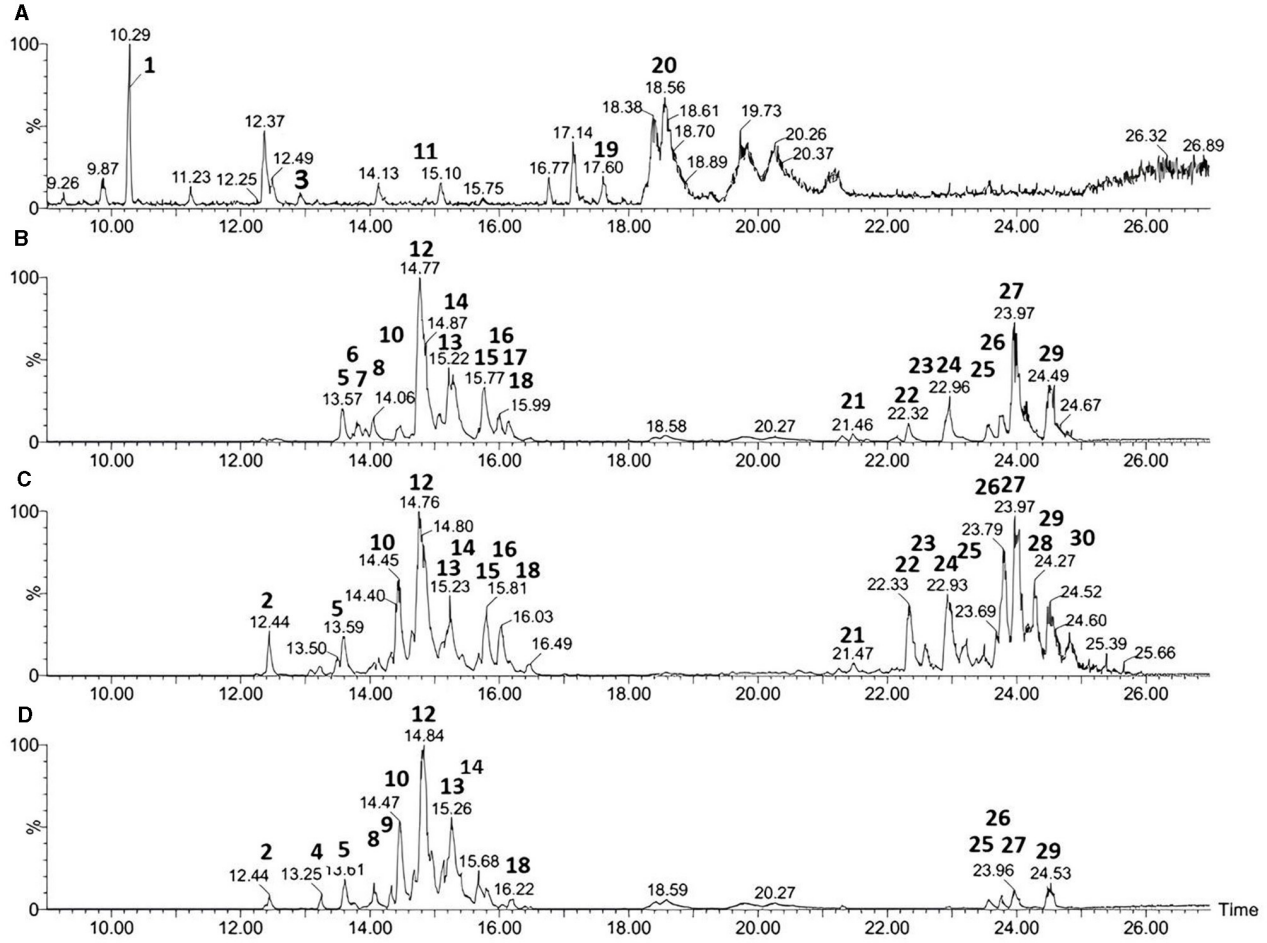
UPLC-ESI-QTOF-MS chromatograms of lipopeptides produced by *Paenibacillus* LIS04 **(A)** and *Bacillus* CT02 **(B)**, LIS05 **(C)**, IM14 **(D)**. The figure the pics correspond to: Lipopeptide ND (1, 3, 11, 19 and 20); Iturin (2 and 4); Fengycin A (5, 6, 7, 8, 9, 10, 12, 13, and 17); Fengycin B (14, 15, 16, and 18); and Surfactin (21–30).

The results of the UPLC-ESI-QTOF-MS analyses for the three strains of *Bacillus velezensis* and *Paenibacillus ottowii* are shown in Table 2 and Figure 2. The samples of the LIS04 of *Paenibacillus ottowii* showed five significant peaks corresponding to unknown lipopeptides (Table 2, Figure 2A), which were not previously described. Among the 20 metabolites produced by CT02, 12 were fengycin (eight Fen A and four Fen B) and eight surfactin (Figure 2B). The isolate CT02 did not express the lipopeptide iturin. Concerning isolate LIS05, the 19 metabolites corresponded to one iturin (iturin A2), eight fengycin (four Fen A and four Fen B), and 10 surfactin (Figure 2C). Of the 14 metabolites of the strain IM14, two corresponded to iturin (iturin A2 and A3-A5), eight fengycin (six Fen A and two Fen B), and four surfactin (Figure 2D).

**Table 2:** Chemical characterization of lipopeptides from *Bacillus* and *Paenibacillus* species by UPLC-QTOF-MS: a = LIS04; b = CT02; c = LIS05; d = IM14.

**Peak**	**RT (min)**	**Compounds**	CFS^*^	**Molecular formula**	**Ion *m/z***	**Error (ppm)**	**Fragment ions *m/z***	**References**
1	10.29	Lipopeptide ND^*^-1	a	C_47_H_63_N_8_O_7_	851.4822 [M + H]^+^	0.2	437; 409	–
2	12.44	Iturin A2	c,d	C_48_H_74_N_12_O_14_	1,043.5519 [M + H]^+^	−0.7	299; 801; 915	Ye et al., 2012
3	12.95	Lipopeptide ND-2	a	C_47_H_75_N_10_O_11_	955.5617 [M + 2H]^+^	−0.9	478; 353	–
4	13.25	Iturin A3–A5	d	C_49_H_76_N_12_O_14_	1,057.5995	1.2	–	Souza et al., 2018
5	13.59	C_14_ Fengycin A	b, c, d	C_70_H_107_N_12_O_20_	718.3788 [M + 2H]^+^	1.7	966; 1,080	Souza et al., 2018; Villegas-Escobar et al., 2018
6	13.79	C_16_ Fengycin A	b	C_72_H_114_N_12_O_20_	741.3965 [M + 2H]^+^	3.9	966; 1,080	Ma et al., 2016
7	13.94	C_16_Fengycin A	b	C_72_H_114_N_12_O_20_	741.3937 [M + 2H]^+^	0.1	966; 1,080	Ma et al., 2016
8	14.06	C_15_ Fengycin A	b, d	C_71_H_108_N_12_O_20_	725.4003 [M + 2H]^+^	2.3	966; 1,080	Souza et al., 2018; Villegas-Escobar et al., 2018
9	14.33	C_15_Fengycin A	d	C_71_H_108_N_12_O_20_	725.3876[M + 2H]^+^	0.7	966; 1,080	Souza et al., 2018; Villegas-Escobar et al., 2018
10	14.44	C_15_Fengycin A	b, c, d	C_71_H_108_N_12_O_20_	725.3986 [M + 2H]^+^	−3.7	966; 1,080	Souza et al., 2018; Villegas-Escobar et al., 2018
11	14.60	Lipopeptide ND-3	a	C_25_H_44_N_5_O_4_	478.3410[M + H]^+^	3.6	376, 162	–
12	14.79	C_16_Fengycin A	b, c, d	C_72_H_110_N_12_O_20_	732.3915 [M + 2H]^+^	−2.3	966; 1,080	Souza et al., 2018; Villegas-Escobar et al., 2018
13	15.20	C_17_ Fengycin A	b, c, d	C_72_H_110_N_12_O_20_	739.3990 [M + 2H]^+^	3.9	966; 1,080	Souza et al., 2018; Villegas-Escobar et al., 2018
14	15.24	C_16_ Fengycin B	b, c, d	C_74_H_114_N_12_O_20_	746.4111 [M + 2H]^+^	2.9	966; 1,080	Souza et al., 2018; Villegas-Escobar et al., 2018
15	15.79	C_17_ Fengycin B	b, c	C_75_H_116_N_12_O_20_	753.4147 [M + 2H]^+^	3.6	994; 1,180	Souza et al., 2018; Villegas-Escobar et al., 2018
16	16.02	C_16_ Fengycin B	b, c	C_74_H_114_N_12_O_20_	746.4069 [M + 2H]^+^	−2.0	994	Souza et al., 2018; Villegas-Escobar et al., 2018
17	16.14	C_17_ Fengycin A	b	C_72_H_110_N_12_O_20_	739.4049 [M + 2H]^+^	−2.3	966; 1080	Souza et al., 2018; Villegas-Escobar et al., 2018
18	16.42	C_18_Fengycin B	b, c, d	C_76_H_119_N_12_O_20_	760.4244[M + 2H]^+^	1.0	994; 1022; 1180	Ma et al., 2016
19	17.60	Lipopeptide ND-4	a	C_56_H_98_N_16_O_13_	602.4215[M + 2H]^+^	2.3	562; 662	–
20	18.59	Lipopeptide ND-5	a	C_17_H_21_N_2_O_7_	365.1349[M + H]^+^	1.7	163, 105	–
21	21.49	C_14_ Surfactin	b, c	C_45_H_97_N_7_O_19_	1,040.6917	−0.1	685; 909; 1022	Ma et al., 2016
22	22.35	C_12_ Surfactin	b, c	C_43_H_91_N_7_O_18_	994.6500	0.1	685; 873; 976	Tang et al., 2010; Ma et al., 2016
23	22.61	Surfactin	b, c	C_56_H_91_N_7_O_12_	1,054.6807 [M + H]^+^	0.3	441; 554	Ma et al., 2016
24	22.98	Surfactin	b, c	C_51_H_89_N_7_O_13_	1,008.6611	1.4	455; 667; 895	Souza et al., 2018
25	23.58	Surfactin	b, c, d	C_51_H_89_N_7_O_13_	1,008.6600	0.3	455; 667; 895	Souza et al., 2018
26	23.79	Surfactin	b, c, d	C_52_H_91_N_7_O_13_	1,022.6726	−2.6	582; 796; 909	Souza et al., 2018
27	23.97	Surfactin	b, c, d	C_52_H_91_N_7_O_13_	1,022.6714	−3.8	582; 796; 909	Souza et al., 2018
28	24.28	Surfactin	c	C_51_H_89_N_7_O_13_	1,008.6581 [M + H]^+^	−1.6	455; 667; 895	Souza et al., 2018
29	24.51	Surfactin	b, c, d	C_53_H_93_N_7_O_13_	1,036.6929	1.8	441; 685; 923	Souza et al., 2018
30	24.88	Surfactin	c	C_53_H_93_N_7_O_13_	1,036.6910 [M + H]^+^	0.8	441; 685; 923	Souza et al., 2018

ND. not determined.

^*^Cell-free supernatant.

**Figure 3:**
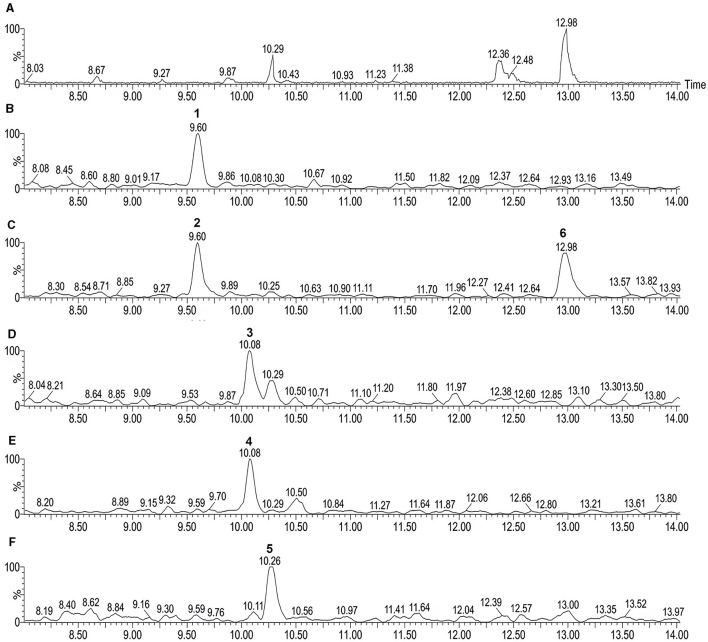
UPLC-QTOF-MS chromatogram of cell-free supernatant (CFS) of *Paenibacillus* LIS04 **(A)**. Expansion of **(A)** showing: **(B)** Fusaricidin C (peak 1); **(C)** Fusaricidin D/E (peaks 2 and 6); **(D)** Fusaricidin A (peak 3); **(E)** Fusaricidin B (peak 4); and **(F)** Fusaricidin A1 (peak 5).

The expansion of UPLC-QTOF-MS chromatogram by searching for its specific *m/z* from the full-scan UPLC-QTOF/MS chromatogram of CFS of LIS04 identified the presence of six fusaricidins C, D/E, A, B, and A1 (Table 3, and Figure 3).

**Table 3:** Chemical characterization of fusaricidins by UPLC-QTOF-MS in CFS of *Paenibacillus otowii* LIS04 based on the reference Qiu et al. (2019).

**Peak**	**Retention time (min)**	**Compounds**	**Molecular formula**	**Ion *m/z***	**Error (ppm)**	**Fragment ions *m/z***
1	9.60	Fusaricidin C	C_45_H_74_N_10_O_12_	947.5598	3.4	Low intensity
2	9.60	Fusaricidin D/E	C_46_H_76_N_10_O_12_	961.5713	−0.9	Low intensity
3	10.08	Fusaricidin A	C_41_H_74_N_10_O_11_	883.5664	5.0	Low intensity
4	10.08	Fusaricidin B	C_42_H_77_N_10_O_11_	897.5738	−3.9	Low intensity
5	10.26	Fusaricidin A1	C_40_H_72_N_10_O_11_	869.5461	0.1	Low intensity
6	12.98	Fusaricidin D/E	C_46_H_76_N_10_O_12_	961.5714	−0.8	Low intensity

### 3.4 Data processing and chemometric analysis

In the PLS-DA analysis, the samples were classified according to the minimum inhibitory concentration values capable of reducing at least 50% of the growth of *Fusarium verticillioides*. Thus, the lower the inhibitory concentration of CFS, the greater the antifungal action. In this sense, the CFS from bacteria of the genus *Bacillus* showed the best performance, emphasizing the strain CT02 grown in a TSB medium.

The 3D PLS-DA score plot represents 75.7% of the total variance explained by three latent variables (LV1 × LV2 × LV3), resulting in greater separation between groups of bacteria, especially between *Bacillus* species (Supplementary Figure S1A). For minimizing overfitting errors, cross-validation was applied using the leave-one-out cross-validation (LOOCV) model, in which cumulative values of *R*^2^ = 0.97676 and Q2 = 0.84549 indicated a three-component model as ideal (Supplementary Figure S1B). Generally, the LV1 axis explains 31.8% of the observed variance, marking the grouping of *Bacillus* samples in negative values without overlap. At the same time, the *Paenibacillus* species was arranged in positive values of LV1. Examining the LV2 axis (20.4%), the separation of samples of *Bacillus velezensis* (LIS05) (positive values) from the other species (CT02 and IM14; negative values).

**Figure 4:**
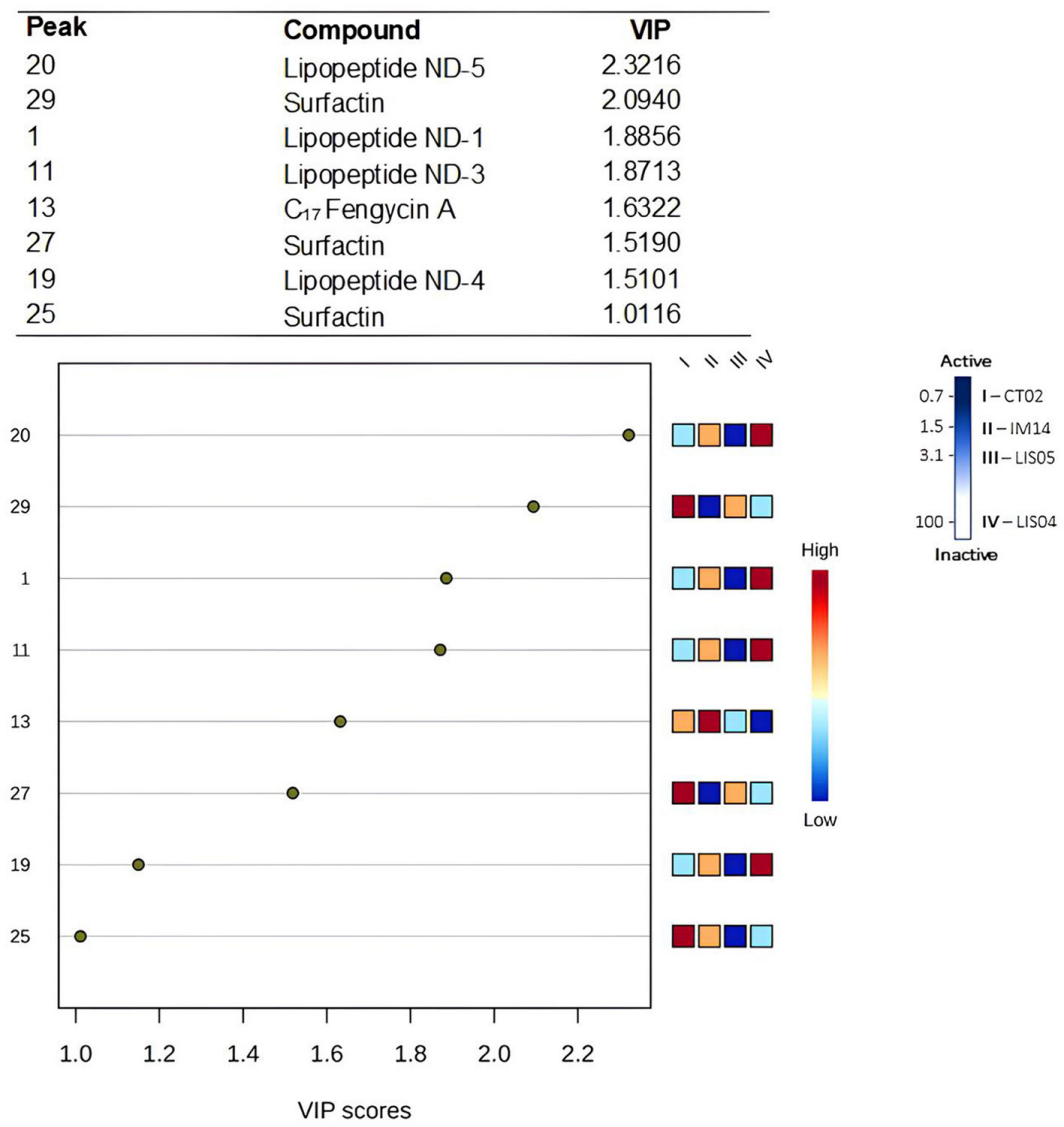
Variable Importance in Projection (VIP) scores for discriminating compounds among the CFS. The higher the VIP score, the better the ability to discriminate between groups. The mini heat map on the right illustrates the relative intensity of the compounds. The samples were classified in ascending order of the minimum inhibitory concentration value, with the lowest values considered more active (represented by the intense blue color on the activity scale). CT02, IM14 and LIS05: *Bacillus* sp., LIS04: *Paenibacillus* sp.

The construction of a PLS-DA model revealed which compounds were most relevant for separating the samples, according to the minimum inhibitory concentration, employing the VIP analysis (Figure 4). Thus, the eight compounds that contributed the most to antifungal activity (VIP > 1.0) could be identified, four of which were more expressive in *Bacillus velezensis* CFS, including surfactin 29 (VIP = 2.094), with higher intensity observed in the CFS of the strain CT02 of *Bacillus* v*elezensis* cultivated in TSB medium. Surfactin (29, 27, and 25) and fengycin A (13) were mainly discriminated against *Bacillus*'s more significant antifungal CFS. For *Paenibacillus ottowii*, the peaks 20, 1, 11, and 19, corresponding to unknown lipopeptides, showed VIP scores above 1.

### 3.5 Genetic and genomic analysis

The genomes of isolates CT02, IM14, LIS05, and LIS04 sequenced on the Illumina HiSeq 4000 platform (Illumina, San Diego, CA, USA) generated a total of 13,573,106, 13,654,038, 13,605,486, and 13,625,202 reads, respectively. All reads were assembled to an initial genome of 4,013,253 bp with 486-fold coverage for CT02, 3,997,574 and 489-fold coverage for IM14, 4,013,450, and 486-fold coverage for LIS05, 5,561,002-fold coverage for 347 for LIS04, using SOAPdenovo v1.05. The G+C content was 46.54%, 46.38%, 46.54%, and 45.43% for CT02, IM14, LIS05, and LIS04, respectively (Supplementary Table S1).

The taxonomic position of strains LIS05, CT02, IM14, and LIS04 was determined by their complete genome sequences using the type (strain) Genome Server (TYGS) platform (Meier-Kolthoff and Göker, 2019). The results showed great proximity of CT02, IM14, and LIS05 with *Bacillus velezensis* strain FZB42. However, the three isolates belong to different strains, and strain LIS04 belongs to the species *Paenibacillus ottowii*, which is highly similar to the type strain MS2379 of *P. ottowii* described by Velazquez et al. (2020). The genes involved in cyclic polypeptide synthesis were identified in each genome (Table 4). The presence of the antifungal gene clusters, coding for the production of non-ribosomal lipopeptides fengycins (*fenABCDE*), surfactins (*srfABCD*), and iturins (*ituABCD*) were confirmed in the three *Bacillus velezensis* strains. The Blastn program (GenBank) showed that the genes involved in synthesizing different lipopeptides antifungal compounds presented variation in nucleotide sequences, ranging from 92 to 100% similarity with previously described genes. For *Paenibacillus* ottowii, the fusaricidin biosynthetic gene cluster (*fusG, fusF, fus E, fusD, fusC, fusB, fusA*, and *fusTE*) involved in the synthesis of fusaricidin was identified in the genome of LIS04 (Table 4).

**Table 4:** Genes involved in the synthesis of bioactive metabolites found in the genome of isolates of *Bacillus velezensis* IM14, CT02, and LIS05 and *Paenibacillus ottowii* LIS04.

**Gene**	**Metabolite**	**Function**	**Genbank similarity (%)**			**Genbank reference**
			***Bacillus velezensis*** **strains**			
			**CT02**	**IM14**	**LIS05**	
*fenB*	Fengycin	Antifungal Induced systemic Resistance (ISR)	97.94	97.72	97.94	MT386612.1
*fenA*			92.61	92.61	92.61	AB973486.1
*fenC*			97.65	98.12	97.65	KU504270.1
*fenD*			100.0	99.18	100.0	OX460973.1
*fenE*			99.91	99.40	99.91	OX460973.1
*srfAA*	Surfactin	Antifungal Induced systemic Resistance (ISR)	98.12	98.24	98.12	CP040881.1
*srfAB*			98.02	97.98	98.02	CP040881.1
*srfAC*			97.86	97.86	97.86	CP040881.1
*srfAD*			99.18	99.18	99.18	CP040881.1
*ituD*	Iturin	Antifungal	96.51	96.67	96.51	KT781919.1
*ituA*			98.11	97.53	98.11	KT781919.1
*ituB*			96.76	96.79	96.76	KT781919.1
*ituC*			97.18	97.13	97.18	KT781919.1
			***Paenibacillus ottowii*** **strain**			
			**LIS04**			
*fusTE*	Fusaricidin	Antifungal	87.24			CP025957.1
*fusA*			88.88			CP025957.1
*fusB*			87.50			CP025957.1
*fusC*			88.08			CP025957.1
*fusD*			91.91			CP025957.1
*fusE*			91.91			CP025957.1
*fusF*			90.06			CP025957.1
*fusG*			89.23			CP025957.1

## 4 Discussion

### 4.1 *In vitro* tests

The growing search for sustainable agriculture, with a reduction in the use of pesticides, has driven the interest in discovering beneficial microorganisms with multiple functions that can contribute to the health and growth of plants. In this study, we tested two *Bacillus velezensis* (CT02 and IM14) previously isolated from maize and one *Bacillus velezensis* (LIS05) and *Paenibacillus ottowii* (LIS04) from sorghum, which inhibited the growth of *F. verticillioides in vitro* (Diniz et al., 2022, 2023). These groups of bacteria produce many compounds with antifungal activity, mainly cyclic lipopeptides.

Many factors can influence bacterial secondary metabolite production and antagonistic activity (Ahsan et al., 2022; Abdul Hakim et al., 2023). Thus, many biosynthesis genes remain silent, and such “cryptic” or “orphan” pathways are only activated under specific conditions (Scherlach and Hertweck, 2009). However, metabolomics can only identify expressed metabolites. Thus, the study's success relies on maximizing the gene expression of a targeted organism under study (Walsh and Fischbach, 2010; Covington et al., 2017). Some studies have suggested that up to 90% of secondary metabolite gene clusters remain silent under standard growth conditions (Walsh and Fischbach, 2010; Covington et al., 2017). Thus, we tested different carbon sources to determine the best medium for metabolite production. The CFS of each bacterial isolate showed variable inhibitory effects on the growth of *F. verticillioides*. For example, the CFS of the two *Bacillus velezensis* strains (CT02 and IM14) showed the highest antifungal activity in TSB and Landy media. This result confirmed the findings of several authors who demonstrated that the medium formulation could increase or suppress the bacterial production of bioactive compounds (Borowicz and Omer, 2000; Peighami-Ashnaei et al., 2009; Kilani-Feki et al., 2016; Sun et al., 2019; Abdul Hakim et al., 2023). However, Sarwar et al. (2018) and Kilani-Feki et al. (2016) reported that CFS of *Bacillus* species grown in LB and M13 media also effectively augment the production of antifungal molecules against different fungal pathogens. Thus, the medium composition may also affect the amount of specific compounds a bacterium produces. Sun et al. (2019) observed the effect of the culture medium on the production of bioactive compounds by *Bacillus natto*. These authors showed that the maximum production of iturin and surfactin was obtained in the Landy medium and potato dextrose broth (PDB), respectively. In our study, the CFS of the three strains of *Bacillus velezensis* grown in TSB and Landy medium were more effective for inducing antifungal activity against *F. verticillioides*. For *Paenibacillus ottowii*, regardless of the four culture media, a CFS concentration of 100% was necessary to inhibit at least 50 % of the *F. verticillioides* growth.

According to our previous reports (Diniz et al., 2021), the visual analysis of SEM images further underscores the inhibitory effect of the cell-free supernatant (CFS) on the fungal hyphae, the inhibition of spore germination and the normal development of *F. verticillioides*. In addition, Huang et al. (2015) in their study found that the CFS of the endophytic culture of *Bacillus atrophaeus* XW2 was hostile to the development of hyphae and the germination of spores of *Colletotrichum gloeosporioides*. These findings imply that antifungal elements in the CFS, such as cyclic lipopeptides and lytic enzymes, can stop hyphal progression. It is known that the mycelium is the vegetative structure that plays a fundamental role in asexual reproduction and disease progression. Therefore, damage caused to the integrity of this structure can reduce pathogenicity and prevent the establishment of a phytopathogenic fungus (Borah et al., 2016).

### 4.2 Metabolite identification based on UPLC-MS analysis

Identifying the bacterial molecules with antifungal activity is an essential step for determining the potential role of each molecule individually. This procedure is also crucial, as it allows the identification of genes and metabolic pathways involved in their synthesis, which is a crucial step for metabolic pathway engineering. Furthermore, identifying bioactive molecules enables chemical synthesis for the commercial development of biofungicides. Different approaches, including gas chromatography or liquid chromatography hyphenated with mass spectrometry techniques, metagenome, and genome-mining, can identify bioactive molecules. In addition, these approaches increase the probability of finding new bioactive molecules. In the present study, the samples of the four strains were analyzed by UPLC-MS to identify the bioactive molecules involved in the antifungal activity as widely described in the literature (Beatty and Jensen, 2002; Tendulkar et al., 2007; Passera et al., 2017; Sass et al., 2021; Xiong et al., 2022). Two *Bacillus velezensis* strains (LIS05 and IM14) secreted fengycin A and B, surfactin, and iturin in the culture medium, while the isolate CT02 secreted fengycin and surfactin but not iturin. It is important to emphasize that while the two iturin-producing strains (LIS05 and IM14) were cultured in the Landy medium, the isolate CT02, which did not produce iturin, was cultured in the TSB medium. Thus, it is reasonable to assume that some isomers of biosynthetic pathways of iturin were not expressed *in vitro* since they were not observed in the UPLC-MS analysis. Concerning *Paenibacillus* sp., the strain LIS04 produced fusaricidins ABCDE and five undetermined lipopeptides.

The chemometric analysis showed that the CFS from bacteria of the genus *Bacillus* showed the best performance according to the values of minimum inhibitory concentration reduced, at least, 50% of the growth of *Fusarium verticillioides*. Also, bacterial antifungal activity may result from different molecules acting synergistically. Otherwise, UPLC-MS-based metabolic fingerprints represent only part of the chemical constituents since a myriad of other bacterial compounds were not covered by this approach, e.g., extracellular lytic enzymes such as chitinase, cellulase, glucanase and protease, siderophores, antibiotics, nitric oxide dioxygenase, and volatile organic compounds (VOCs), which had strong inhibitory effect fungal growth (Petatán-Sagahón et al., 2011; Diniz et al., 2021, 2022; Soliman et al., 2022).

Species of *Bacillus* and *Paenibacillus* are of particular biotechnological interest because they produce many biologically active secondary metabolites that regulate various diseases and stimulate plant growth. However, these compounds are produced only in response to external stimuli, such as nutrient sources, primarily carbon, or under specific conditions like the stationary growth phase (Malik, 1980; Ruiz et al., 2010). Thus, variations in metabolite production are frequently observed within the same strain or between strains of the same species cultivated *in vitro* (Tulp and Bohlin, 2005; Winter et al., 2011). It is also important to emphasize that only a fraction of the biosynthetic capabilities suggested by genomic analyses have been observed under laboratory conditions because the expression of many, perhaps even most biosynthetic pathways, depends strongly on the environmental conditions (Forseth et al., 2012).

Sequencing complete genomes of antagonists and plant growth-promoting microorganisms may be used to identify functional genes not expressed *in vitro*. This strategy has enabled the confirmation and discovery of new antimicrobial genes and improved the effectiveness of these biological control agents (Palmieri et al., 2022). Thus, to clarify this possibility, we performed the whole-genome sequencing of the four strains to confirm these aspects of metabolite production *in vitro*.

### 4.3 Genome analysis

The genus *Bacillus* consists of many species with a high degree of similarity. The genome analysis of the three *Bacillus* strains demonstrated that they belong to the *Bacillus velezenzis* species of the *Bacillus amyloliquefaciens* group, composed of *B. amyloliquefaciens, B. velezensis, B. nakamurai*, and *B. siamensis* (Wang et al., 2022). Since the *Bacillus amyloliquefaciens* group is formed by closely related species, separating them by traditional taxonomy is challenging. In addition, molecular data from partial sequencing of the 16S rRNA gene alone or associated with housekeeping genes, such as atpD, gyrB, recA, rpoB, and trpB, also shows unsatisfactory results for distinguishing *B*. *velezenzis* from its peer due to the highly conserved nature of these genes in this group. Complete genome sequencing emerged as a powerful tool for correctly associating bacterial species (Rooney et al., 2009). With the increasing number of complete genome sequencing of bacterial strains and comparative genomic analysis, a clear identification of species, subspecies, and strains has augmented rapidly (Liao et al., 2023).

The genetic analysis of strains CT02, IM14, and LIS05 using the complete genomic sequences showed that the three strains are closer to the FZB42 strain *B*. *velezensis* of the *B. amyloliquefaciens* group. Recently, these species have been used as biological control agents in agriculture (Borriss, 2011). Among the various molecules with antifungal activity produced by *Bacillus velezenzis*, three cyclic lipopeptides, surfactin, iturin, and fengycin, are the most effective against fungi. The lipopeptides are characterized by their amphiphilic nature; these molecules disrupt the membrane structures of filamentous fungi. The genomic analysis of the three strains confirmed the presence of genes involved in lipopeptide synthesis with antifungal activity identified by HPLC-MS (Table 4).

Although the iturin lipopeptide was not detected by UPLC-MS in the isolate CT02, the genome analysis revealed that the ORFs of the four genes of the iturin cluster were intact. It is well known that many factors affect the production of Iturin homologs (A2–A6) by *B*. *subtilis* (Mizumoto and Shoda, 2007; Noriyasu et al., 2009; Yue et al., 2021). The concentrations of carbon and nitrogen sources, as well as concentrations of precursor amino acids l-asparagine (Asn), l-aspartic acid (Asp), l-glutamic acid (Glu), l-glutamine (Gln), l-Serine (Ser) and l-proline (Pro) are critical factors affecting iturin production by *Bacillus*, and minor factors, such as pH, temperature, relative humidity, and volume of inoculum, influence iturin production (Mizumoto and Shoda, 2007).

In our study, the samples submitted to UPLC-MS analysis were obtained from different culture media based on the lowest inhibitory concentration of the fungus. The concentration of sucrose in the TSB medium containing 2.5 g.L^−1^ sucrose used for CT02 and 20 g.L^−1^ in the other media may partially explain that CT02 does not produce sufficient iturin to be detected by UPLC-MS. This result also suggests that other molecules with antifungal activity were present in the cell-free supernatant of CT02.

This result highlighted that only a fraction of the biosynthetic capabilities suggested by genomic analysis has been observed under laboratory conditions because the expression of many, perhaps even most biosynthetic pathways, depends strongly on environmental conditions (Forseth et al., 2012).

The genomic analysis of LIS04 revealed that this strain was highly similar to the strain *P*. *ottowii* MS2379. Previous studies using the 16S rRNA and rpoB gene analysis have assigned the strain MS2379 with *P*. *polymyxa* (DSM 36 T), *P*. *jamilae* (DSM 13815 T), and *P*. *peoriae* (DSM 8320 T). Also, studies using the 16S rRNA and rpoB gene analysis have assigned the strain MS2379 with *P. polymyxa* (DSM 36 T), *P. jamilae* (DSM 13815 T), and *P. peoriae* (DSM 8320 T). However, in a study by Velazquez et al. (2020) using physiological, chemotaxis biochemical, and nucleic acid hybridization, the strain MS2379 was proposed as a new species named *P. ottowi*. In this context, our study is the first report using *Paenibacillus ottowii* for controlling *Fusarium*, identifying five lipopeptide candidates for antifungal activity and the genes responsible for fusaricidin synthesis.

The genomic analysis substantiated the UPLC-MS data confirming the existence of genes for secondary metabolites with broad-spectrum antifungal activities (Ongena and Jacques, 2008; Sood et al., 2014), that is, the clusters of lipopeptides iturin, fengycins, and surfactins in the three strains of *Bacillus velezensis* and fusaricidin and five not-identified lipopeptides in LIS04 *Paenibacillus ottowii*.

## 5 Conclusions

The present study showed relevant results concerning bacterial antifungal activity *in vitro* and the identification of antifungal lipopeptides for controlling *Fusarium verticillioides* in maize crops. The genome sequencing revealed the taxonomic position of the four strains (three *B*. *velezensis* and one *P*. *ottowii*), and genome mining showed the antifungal gene clusters of non-ribosomal lipopeptides. This is the first study exploring the antifungal activity of *Paenibacillus ottowii*, and UPLC-MS analysis showed five new unknown lipopeptides in this species. A multidisciplinary approach may reveal the structure of the five new lipopeptides of *P*. *ottowii* and the identification of their genes. Biological tests with the purified lipopeptides will show their potential use in agriculture. The four strains in this study have great potential for developing new bioproducts to protect plants against *F*. *verticillioides*, promote sustainable development, and contribute to environmental preservation by reducing the use of fungicides in agriculture.

## Data Availability

The data presented in the study are deposited in the repository NCBI https://www.ncbi.nlm.nih.gov/genbank/ with accession numbers JAUUUW000000000, JAUUUY000000000, JAUUUZ000000000, and JAUUUV000000000.
