# Supplementary material for: Chemical and genetic characterization of lipopeptides from Bacillus velezensis and Paenibacillus ottowii with activity against Fusarium verticillioides
Source: Front Microbiol. 2024 Aug 26;15:1443327. doi: 10.3389/fmicb.2024.1443327 (PMC11381237; doi:10.3389/fmicb.2024.1443327)
Supplement: Supplementary file 1 [file Table_1.DOCX]

Supplementary Material


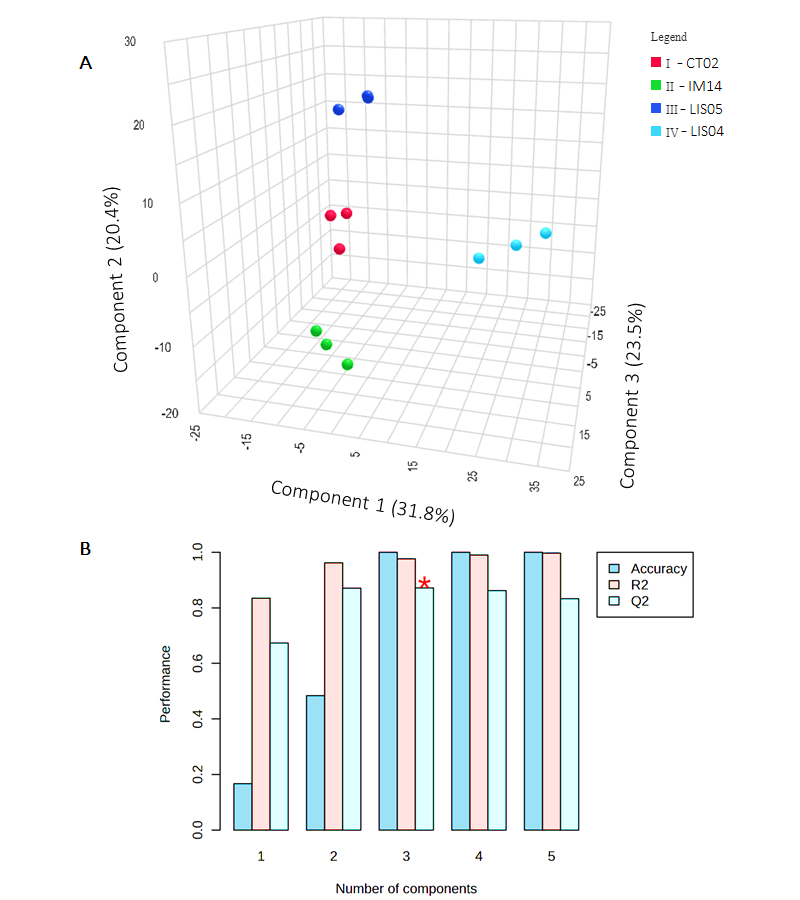


**Figure S1** - 3D PLS-DA scores based on UPLC-MS dataset of the CFS. Discrimination was characterized by 31.8% for LV1, 20.4% for LV2, and 23.5% for LV3 (A). Cross-validation graph using the leave-one-out cross-validation (LOOCV) method applied to partial least squares discriminant analysis (PLS-DA) data. The red star above the Q2 column indicates that the three-component model is optimal (B). CT02 (I), IM14 (II), LIS05 (III) and LIS04 (IV).

**Table S1 –** General genomic characteristics of three strains *Bacillus velezensis* CT02, IM14 LIS05, and *Paenibacillus ottowii* LIS04.

| Assembly | *Bacillus* CT02 | *Bacillus* IM14 | *Bacillus* LIS05 | *Paenibacillus* sp. LIS04 |
| --- | --- | --- | --- | --- |
| # Contigs (>=0 bp) | 30 | 57 | 30 | 212 |
| # Contigs (>=1000 bp) | 11 | 24 | 11 | 47 |
| Total length (>=0 bp) | 4013253 | 3997574 | 4013450 | 5561002 |
| Total length (>=1000 bp) | 4007327 | 3985460 | 4007524 | 5510934 |
| # Contigs | 28 | 57 | 30 | 212 |
| Largest contig | 1354340 | 713769 | 1354537 | 931586 |
| Total length | 4013253 | 3997574 | 4013450 | 5561002 |
| GC (%) | 46.54 | 46.38 | 46.54 | 45.43 |
| N50 | 1072201 | 468182 | 1072201 | 623597 |
| N75 | 642316 | 236038 | 642316 | 222165 |
| L50 | 2 | 4 | 2 | 4 |
| L75 | 3 | 7 | 3 | 8 |
| # Total reads | 13573106 | 13654038 | 13605486 | 13625202 |
| # Left | 6786553 | 6827019 | 6802743 | 6812601 |
| # Bright | 6786553 | 6827019 | 6802743 | 6812601 |
| Mapped (%) | 98.97 | 98.98 | 98.97 | 98.78 |
| Properly paired (%) | 97.67 | 97.23 | 97.53 | 96.68 |
| Avg. coverage depth | 486 | 489 | 486 | 347 |
| Coverage >= 1x (%) | 100.0 | 100.0 | 100.0 | 100.0 |
| N's per 100 kbp | 2.39 | 2.28 | 2.39 | 1.76 |
